# Ecuadorian Holstein-Friesian cattle paternal lineages: Demographic structure, inbreeding evolution, and genetic diversity

**DOI:** 10.1371/journal.pone.0318730

**Published:** 2025-02-25

**Authors:** Luis F. Cartuche-Macas, Miguel A. Gutierrez-Reinoso, Edilberto Chacón, Carlos O. Larrea-Izurieta, Joar M. García-Flores, Manuel Garcia-Herreros

**Affiliations:** 1 Instituto de Investigación de la Biodiversidad “Pachamamata Kamak”, Universidad Intercultural de las Nacionalidades y Pueblos Indígenas (UINPIAW), Quito, Ecuador,; 2 Asociación Holstein Friesian del Ecuador (AHFE), Quito, Ecuador; 3 Facultad de Ciencias Agropecuarias y Recursos Naturales, Carrera de Medicina Veterinaria, Universidad Técnica de Cotopaxi (UTC), Latacunga, Ecuador; 4 Facultad de Ciencias Veterinarias, Departamento de Ciencia Animal, Laboratorio de Biotecnología Animal, Universidad de Concepción (UdeC), Chillán, Chile; 5 Escuela Superior Politécnica Agropecuaria de Manabí Manuel Félix López (ESPAM), Carrera de Medicina Veterinaria, Calceta, Ecuador; 6 Facultad de Medicina Veterinaria y Agronomía, Carrera de Medicina Veterinaria, Universidad UTE, Quito, Ecuador; 7 Instituto Nacional de Investigação Agrária e Veterinária, Santarém, Portugal; 8 CIISA-AL4AnimalS, Faculty of Veterinary Medicine, University of Lisbon, Lisbon, Portugal; National Bureau of Animal Genetic Resources, INDIA

## Abstract

Holstein-Friesian cattle are the most important dairy breed worldwide. The main objective was to carry out a detailed pedigree evaluation of the Ecuadorian Holstein-Friesian cattle to investigate the demographic structure, inbreeding evolution, and genetic diversity of the official paternal lineages to determine the potential GD loss after the inclusion of modern Assisted Reproductive Technologies (ARTs). Official pedigree information from 28,893 Holstein-Friesian sires born between 1950 and 2021 and enrolled with the Ecuadorian Holstein-Friesian Association (AHFE, Ecuador) were recorded and evaluated from USA and Canada genetic official databases. After multiple-trait across-country genetic analyses the total population was divided into four subpopulations; i) 1950–1969: natural mating (NM) period; ii) 1970–1989: NM +  artificial insemination (AI) period; iii) 1990–2009: AI +  embryo transfer (ET) period; and iv) 2010–2021: AI +  ET +  genomic selection (GS) period. Demographic parameters [number of males, pedigree integrity (PI), and generation interval (GI)] were analysed. PI was analysed using known ancestors up to 4 generations considering the number of complete (GCom), maximum (GMax) and equivalent (GEqu) generations. Moreover, Inbreeding parameters [inbreeding coefficient (F), average relatedness (AR), coancestry (C), effective size (Ne), genetic conservation index (GCI)] and parameters related to the gene origin probability (number of founders (f), effective number of founders (fe), genetic conservation index (GCI), among others] were also analysed, together with the fe/f ratio, fge/fe ratio, genetic contributions, and genetic diversity loss (GD-loss) derived parameters. The results indicated that nearly all imported sires used in Ecuador born in the beginning of 1990s could be traced to just three countries, who together account for > 90% of paternal lineages. This fact indicates that GD has undergone a dramatic decrease during the past 30 years. The PCI for the three last periods were > 55%, and the trend was enhanced in the fourth chronological period till > 92%. The estimated proportion of random genetic drift in GD loss increased over time as well as the Ne that decreased by the time. In conclusion, the occurrence of AI +  ET +  GS period led to the major GD loss. Therefore, due to the extremely limited number of paternal lineages the strategy for recovering the minimal GD on the current and future Ecuadorian Holstein-Friesian cattle should reduce the inbreeding values by increasing the Ne using alternately the foreign genetic material and the national breeding stock.

## Introduction

The Holstein-Friesian cattle are the world´s most widespread breed for milk production [1]. Inbreeding depression is a major problem in the more specialised breeds where the massive genetic progress achieved in recent generations is characterized by a dramatic genetic diversity (GD) loss that can cause animal health problems [2,3]. The increase of dairy productivity has resulted in a drastic reduction of GD due to intensive sire selection [4,5,6]. In recent decades, many breeding programmes have been based on a few paternal lineages with high heritable transmission of the most important productive traits, such as milk volume, fat and protein percentage, longevity, and fertility [2,7,8]. The Holstein-Friesian-derived genotype was introduced in Ecuador during the first decade of the 1900s and the official registration by the Ecuadorian Holstein Friesian Association began in 1948 [9,10]. Thus, the genetic improvement of Ecuadorian Holstein-Friesian breed has been based mainly on the importation of sires, semen straws and embryos, whose genetic evaluation has been previously carried out in the countries of origin [11]. In Ecuador, the use of artificial insemination (AI) began in 1952; however, it was not totally consolidated till the early 1970s [9,10]. With the advent of AI, semen imports increased (mainly from the USA and Canada) along with a reduction in the imported sires [12]. On the other hand, the research from Henderson et al. [13] in the 1960s using the BLUP methodology for genetic evaluation together with the genomic selection in the last decade [14] has resulted in a small group of genetically superior sires which have been used on a massive scale based on their genetic merit by the so-called ‘Multiple Across Country Evaluation’ (MACE; e.g., milk production, udder health, conformation, longevity, calving ease and fertility, among others) in different countries [5]. This fact together with the progress of Assisted Reproductive Technologies (ARTs) has resulted in a faster expansion of the Holstein-Friesian cattle in different South American countries, including Ecuador [15]. In Ecuador several ARTs such as multiovulation and embryo transfer (MOET) were consolidated at the end of the 1990s and the *in vitro* fertilization technologies in the mid-2000s [16]. These factors have led to significant genetic gains in traits of economic importance within populations but have also affected the GD of individuals in some countries [17,18,19,20]. The GD of bovine populations has been studied through genealogical and molecular data in many countries and it has been reported that parameters such as inbreeding coefficient (F) was increased while effective population size (Ne) and generation interval (GI) decreased, accelerating the GD loss [21,22,23]. The decrease in GD is mainly due to the inbreeding increase which enhances the probability of occurrence of negative traits caused by recessive alleles [24]. A clear example of this phenomenon in the Holstein-Friesian breed is the occurrence of recessive pathologies affecting the production and important economic traits within certain paternal lineages [25,26]. In the last decade, the study of GD from the Y-chromosome has gained importance due to the limited number of ancestors within the Holstein-Friesian and Jersey breeds [8]. For example, it has been shown that the oldest ancestors present in the current US Holstein-Friesian population were Hulleman and Neptune H [8]. Moreover, in the Jersey breed the oldest ancestors were Secret Signal Observer and Advancer Sleeping Jester [27]. In addition the absence of homologous recombination in a specific region called MSY for the Y-chromosome at meiosis has led to a reduction in the Ne [28,29]. In particular, in cattle, studies of genes within the Y-chromosome were related to male phenotypic traits, spermatogenesis and fertility [30].

In Ecuador there are very few studies on GD and ancestry in the Holstein-Friesian breed [31]. Therefore, in order to gain insight into the effects of paternal lineages on the GD, the main objective of the present study was to analyse the demographic structure, inbreeding evolution, and genetic diversity of the official paternal lineages to determine the potential GD loss after the inclusion of modern Assisted Reproductive Technologies (ARTs). For this purpose, official genealogical information registered was used to determine the GD of the current population based on information obtained from imported paternal ancestors and national sires born and registered between 1950 and 2021. This study will generate base information to establish conservation strategies and reduce the effects of the GD loss in the populations of the Holstein-Friesian cattle breed in Ecuador.

## Materials and methods

### Ethical statement

This research was performed under the Project License PRO-INV-001-2023 entitled “Population structure and genetic diversity in Ecuadorian Holstein-Friesian cattle” from the Ecuadorian Holstein-Friesian Association (EHFA, Ecuador). The present research did not require any animal handling, since the study was directly carried out using the records and databases provided by the EHFA, the National Association of Animal Breeders (NAAB, USA), the Holstein Association of Canada and the Canadian Network for Dairy Excellence (CNDE, Canada).

### Study area

The distribution of the Holstein-Friesian breed population was monitored yearly by a field study performed by the Ecuadorian Government and the EHFA (Quito, Ecuador). Most of the Ecuadorian Holstein-Friesian population was located in the central Andean mountain range at an altitude of ~  2000–3,000 m.a.s.l. The Köppen climate classification is BSki, ETH, and Cwb (cold semi-arid highland, tundra-desert highland, and subtropical highland climate, respectively) with mean annual temperature of ~ 9 ±  3.1°C and a relative humidity of ~ 80%. The study included the individual pedigree records from parental lineages of Holstein-Friesian breed in order to have longitudinal information that allows us to perform the present study.

### Holstein-friesian genealogical databases

The pedigree records were obtained from the dairy control database of the EHFA, NAAB, and CNDE. A total of 28,893 sires were analysed, of which 19,799 (68.52%) were born and registered in Ecuador between 1950 and 2021. Information for the flow study and genetic relationships evaluated included: a) sire’s name and code; b) dam’s name and code; c) sire’s identification; and d) date of birth. Missing paternal ancestry information was obtained from the NAAB (https://www.holsteinusa.com/) and the Holstein Association of Canada and CNDE (https://www.cdn.ca/home.php) databases. The data obtained were stored in a Microsoft Excel 2019 file, where each of the paternal ancestors was identified according to the year of birth of all the sires born in each country including Ecuador. The database was divided into four populations: a) sire population between 1950 and 1969 (natural mating (NM) period); b) sire population between 1970 and 1989 (NM +  artificial insemination (AI) period); c) sire population between 1990 and 2009 (AI +  embryo transfer (ET) period); and d) sire population between 2010‒2021 (AI +  ET +  GS period). The different populations were evaluated using descriptive statistics using SPSS v26 Statistics for Windows (IBM Corp., Chicago, IL, USA). Similarly, the ancestry of the sires was analysed in the same four periods as described before according to different studies [8,27,29,32].

### Analysis of demographic-derived parameters

Number of births: The number of births was computed to determine the maximum and the average number of offspring per sire.

Generation interval (GI): The average age of parents at the birth of their offspring and generational intervals were estimated for the four generation pathways: sire-son, dam-son, sire-daughter, and dam-daughter from the date of birth of parents and offspring [33].

Pedigree integrity: Pedigree integrity was assessed through the Pedigree Completeness Index (PCI) in the historical and current population considering the premises of Navas et al. [34]. In addition, pedigree integrity was determined up to the fourth generation of ancestors, the maximum number of generations (GenMax), the common number of generations (GenCom), the equivalent number of generations (GenEqu), and finally, the genetic conservation index (GCI) in the historical and current populations.

### Analysis of genetic-derived parameters for variability assessment

The ancestry contribution in the four populations (sires used in the four chronological periods) was analysed according to James [33]. Gene flow and the net merit index of the imported and national sires were evaluated on the basis of the pedigree record information obtained from the country of origin including their ancestors. Furthermore, the genetic analysis of the sires, the inbreeding coefficient (F) and the average pedigree and genomic relatedness (ΔR) were analysed as well as the effective population size (Ne). The estimation of pedigree quality and genetic variability was performed using the ENDOG v.4.8 software [35]. The analysis of pedigree and genomic inbreeding was also included having into account the information derived from the country of origin of each imported and national sire with the aim of complement the analysis. Regarding genetic parameters were assessed as follows:

Inbreeding coefficient (F): The F was calculated as the probability that both homologous genes found in the same zygote are identical by descent.

Co-ancestry coefficient (C): The C between two individuals was calculated as the probability that genes taken at random from each individual are identical by descent [36].

According to Leroy et al. [37], C and F are estimators of identity-by-descent (IBD) and they differ whether the alleles are considered to belong to one individual (F) or to two individuals (C). This makes the C of two individuals the F of their potential offspring. The methods described by Meuwissen and Luo [38] were used to estimate C and F.

Coefficient of relatedness (AR): The average individual AR refers to the probability that two related individuals have inherited a particular allele of a locus/gene from their common ancestor and was estimated according to Gutierrez et al. [35].

Individual inbreeding rate (∆F): The ∆ F for each generation was calculated according to Gutiérrez et al. [39] according to the following equation:


$$\Delta F_{b}=1-\sqrt[{t_{b}-1}]{1-F_{b}}$$

where, *t*_*b*_ was the number of equivalent generations and F_*b*_ was the F of individual *b*.

Individual coancestry rate (∆C): The ∆ C for each generation was calculated according to the methods described by Cervantes et al. [40]:


$$C_{ba}=1-\sqrt[{\frac{tb+ta}{2}}]{1-C_{ba}}$$

where, *tb* and *ta* were the number of equivalent generations and C_ba_ was C for individuals *b* and *a*.

Effective population size (Ne): The Ne was calculated according to Wright [41] as follows:


$$\bar{N_{e}}=\frac{1}{2\bar{\Delta IBD}}$$

Equivalent subpopulations (S): The number of S was calculated according to Cervantes et al. [42] following the equation:


$$S=\frac{\bar{N_{e}C_{i}}}{N_{e}F_{i}}$$

where, $\bar{N_{e}C_{i}}=\frac{1}{\left(2\bar{\Delta C}\right)}$ was the average Ne computed considering C, and $\bar{N_{e}F_{i}}=\frac{1}{\left(2\bar{\Delta F}\right)}$ was the average Ne computed considering F.

Genetic conservation index (GCI): This GCI was estimated for each individual according to the methodology of Alderson [43], which was based on the assumption that the aim of a conservation programme was to conserve the full range of alleles of the base population to calculate an effective number of founders in the pedigree of an individual. The higher the GCI value the higher the value of an individual for conservation. This index considers the genetic contribution of all founders identified as:


$$GCI=\frac{1}{\sum p_{i}^{2}}$$

where, p_*i*_ was the proportion of genes of founder *i* in the animal’s pedigree.

Effective number of founders (f_e_): The f_e_ is a crucial parameter for analyzing the number of equally contributing funders to generate the same amount of GD in the population. The f_e_ was calculated using the following equation proposed by Lacy [44]:


$$fe=\frac{1}{\begin{array}{c} f\\ \sum fq_{\begin{array}{c} i\\ i=1 \end{array}}^{2} \end{array}}$$

where, *f* is the total number of founders and *q*_*i*_ is the genetic contribution to the *i*th founder to the reference population.

Founder genome equivalent (f_ge_): The f_ge_ is the parameter that shows the number of equally contributing funders with no random loss of founder alleles. The f_ge_ was calculated using the following equation [45]:


$$fge=\frac{1}{2fg}$$

where *fg* is the average con-ancestry for the considered population.

Genetic diversity (GD): The GD was calculated by the following equation proposed by Lacy et al. [44]:


$$GD=1-\frac{1}{2fge}$$

where, f_*ge*_ was the founder genome equivalents.

The resulting value obtained from the 1 – GD expression shows the amount of GD lost in the population.

Genetic diversity loss (GD*): The GD * was calculated using the following equation proposed by Lacy [44]:


$$GD*=1-\frac{1}{2fe}$$

where, f_*e*_ was the effective number of founders.

The difference between GD * and GD shows the diversity loss due to the genetic drift [46].

### Statistical analysis

The database analysis was carried out using ENDOG v. 4.8 [35] and CFC v. 1.0 [21] software by means of which the demographic-derived parameters, genetic diversity indices, and gene origin probability were obtained. All descriptive statistical analyses were carried out using the statistical software package SPSS for Windows® v. 26 (IBM Corp., Chicago, IL, USA) using the database containing the parameters for determination of gene flow, inbreeding, index for net merit and genetic relationships between sires according to country of origin and birth date. General statistical analysis was performed using one-way ANOVA for the analysis of the different chronological periods, the number of generations between sires and their ancestors, and paternal lineages. Pair-wise comparisons of mean value were conducted by Student’s t-test. The level of significance was set at P < 0.05.

## Results

### Chronological evolution of the Holstein-Friesian population

**Fig 1:**
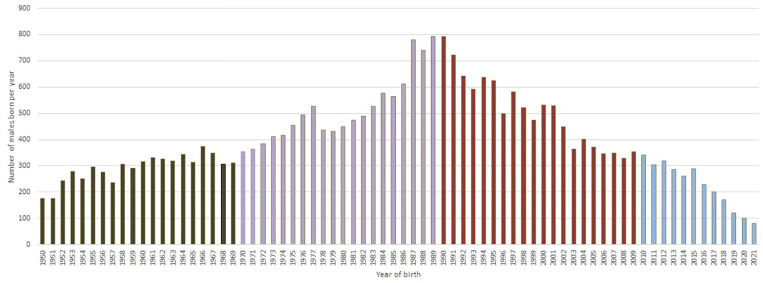
Chronology of the number of Holstein-Friesian males born per year in Ecuador. The registration of purebred bulls has become a routine in Ecuador today by using the EHFA database; however; there still has not been carried out any historical in-depth study of the genetic variability and diversity of the Ecuadorian Holstein-Friesian purebred population. The genetic international influence caused that dairy breeding industry in Ecuador became gradually more developed. Black: NM period (1950–1969); Purple: NM +  AI period (1970–1989); Brown: AI +  ET period (1990–2009); and finally, Blue: AI +  ET +  GS period (2010–2021).

The number of Holstein-Friesian males born per year began to grow significantly from 1950 (NM period) till 1990 (the beginning of the NM +  AI +  ET period). From 1990 onwards the tendency of the population of males born per year diminished till 2021 (AI +  ET +  GS period) (Fig 1).

The highest growth of Holstein-Friesian males born occurred between 1970 and 1990 (NM +  AI period) causing a rapid increase in the total number of sire sons. During the next three decades (1990‒2021), the number of males born diminished drastically as shown by a seven-fold decrease (Fig 1) compared to the end of the second chronological period (NM+AI).

Table 1 shows the top ten most historically influential sires in Ecuadorian Holstein-Friesian cattle population.

**Table 1:** Historical paternal lineages (top ten sires) of Ecuadorian Holstein-Friesian cattle (1990–2021).

N.	Name	ID	Year	Sons born in Ecuador	Fped (%)	Fgen (%)	NM$	TPI (‒)	MLK_ PTA (lb)	FAT_ PTA (lb)	PRO_PTA (lb)	PL_ PTA (mo.)	DPR_PTA (%)
1	VAL-BISSON DOORMAN	HOCAN000107281711	2011	31	5.8	6.3	‒78	2129	‒449	13	10	0	‒25
2	KHW KITE ADVENT-RED-ET	HOUSA000133002953	2001	30	3.6	4.8	‒424	1654	‒1617	‒40	‒43	1	26
3	MAPLE-DOWNS-I G W ATWOOD	HOCAN000008956379	2007	29	8.7	14.0	‒248	1904	‒297	12	‒8	‒26	‒48
4	R-E-W SEAVER-ET	HOUSA000137012381	2005	21	4.7	2.0	‒365	1735	‒781	‒46	‒14	‒9	‒7
5	GEN-MARK STMATIC SANCHEZ	HOUSA000134422312	2003	18	3.9	3.3	‒468	1579	168	‒36	‒7	‒28	‒37
6	PINE-TREE SID-ET	HOUSA000062175895	2005	17	6.8	6.4	‒560	1518	‒838	‒19	‒23	‒39	‒51
7	MOUNTFIELD SSI DCY MOGUL-ET	HO840003006972816	2010	15	6.8	4.4	303	2260	419	40	13	‒2	‒33
8	CRACKHOLM FEVER	HOCAN000103631566	2005	14	5.6	4.3	21	1976	‒397	11	‒11	6	‒7
9	REGANCREST DUNDEE-ET	HOUSA000127640114	1999	13	1.9	4.2	‒665	1375	‒1039	‒56	‒31	‒44	‒3
10	LIRR DREW DEMPSEY	HOUSA000061083609	2005	13	5.6	3.2	‒207	1917	‒731	‒4	‒4	‒19	‒18

Fped: pedigree-based inbreeding; Fgen: genomic-based inbreeding (FROH and FGRM); NM$: Lifetime Net Merit $; TPI: Total Performance Index; and Net Merit (NM$). MLK_ PTA: Predicted transmitting abilities of milk (lb); FAT_PTA: Predicted transmitting abilities of fat (lb); PRO_PTA: Predicted transmitting abilities of protein (lb); PL_ PTA Predicted transmitting abilities of productive life (mo.); DPR_PTA: Predicted transmitting abilities of daughter pregnancy rate (%).

Only two sires belong to the genomic era [VAL-BISSON DOORMAN (2011) and MOUNTFIELD SSI DCY MOGUL-ET (2010)] which in the short term were the most reliable for determining reproductive genetic parameters. The rest of the sires belong to the pre-genomic era (before 2010), which currently have a very high number of daughters, which in the long term makes them highly reliable for determining both reproductive and productive genetic parameters (Table 1).

### Generation intervals

The mean generation intervals (GI) studied in the different chronological periods for the Ecuadorian Holstein-Friesian breed are shown in Table 2.

**Table 2:** Generation intervals (GI) of the sire-son (SS), sire-daughter (SD), dam-son (DS), and dam-daughter (DD) selection pathways by chronological period for the Ecuadorian Holstein-Friesian cattle.

Chronological Period	Generation pathway			
	Sire-son (SS)	Sire-daughter (SD)	Dam-son (DS)	Dam-daughter (DD)
GI (1950‒1969)	6.10 ± 0.08^aA^	5.42 ± 0.04^aA^	4.71 ± 0.06^abA^	4.24 ± 0.05^bA^
GI (1970‒1989)	8.83 ± 0.05^aB^	7.15 ± 0.07^bB^	4.63 ± 0.04^cA^	4.50 ± 0.06^cA^
GI (1990‒2009)	7.93 ± 0.05^aB^	8.82 ± 0.03^aC^	4.61 ± 0.04^bA^	4.84 ± 0.03^bA^
GI (2010‒2021)	4.30 ± 0.09^aC^	7.28 ± 0.04^bB^	3.33 ± 0.06^aB^	4.46 ± 0.04^aA^
Average GI	6.79 ± 0.06^aB^	7.16 ± 0.05^aB^	4.32 ± 0.04^bA^	4.51 ± 0.04^bA^

Generation intervals (GI): a) between 1950 and 1969 (NM period); b) between 1970 and 1989 (NM +  AI period); c) between 1990 and 2009 (AI +  ET period); and d) between 2010‒2021 (AI +  ET +  GS period). Different letters (a–c) within a row show statistical differences generation pathways (p <  0.05). Different letters in a column (A–C) show statistical differences among chronological periods within each generation pathway (p <  0.05).

Gradual increased variations in GI values were observed for the Holstein-Friesian population in all influential selection paths (SS, SD, DS and DD) between 1950 and 1989, being these periods corresponding to the first (NM) and second (NM +  AI) period before the introduction advances ARTs (embryo transfer) in Ecuador (Table 2). Within these periods, the GI value increased by 28.17% (SS), 24.93% (SD), and 12.60% (DD). There was also a stable value in GI in the DS path with a marginal increase of 2.76% within the same period. However, just about a couple decades after the introduction of embryo transfer technologies, GI declined significantly in the male pathway SS and in the female pathway DS in the Ecuadorian Holstein-Friesian population. Thus, between 1989 and 2009 there were 13.87% and 7.89% reductions in GI in the SS and DS paths, respectively (Table 2). Surprisingly, an increase of 15.06% in GI was observed in the male path SD during this period. There was also a stable value in GI in the DD path with a marginal decline of ~ 1% within the same period. Finally, a reduction of the values were observed in all paths between 2009 and 2021 by 49.05% (SS), 18.23% (SD), 24.41% (DS), and 8.40% (DD) corresponding to the genomic selection era in Ecuador (Table 2).

**Fig 2:**
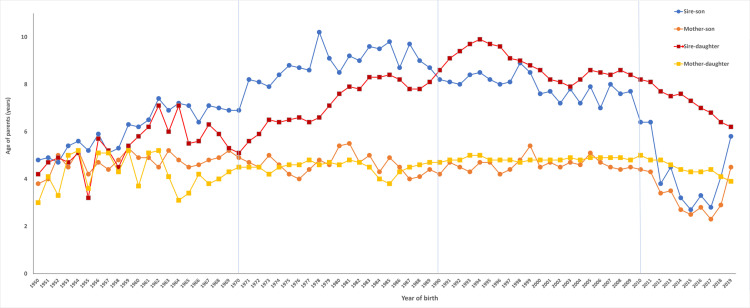
Average age of the sire-son (SS), sire-daughter (SD), dam-son (DS), and dam-daughter (DD) of the different selection pathways in the Holstein-Friesian breed in Ecuador. NM period (1950‒1969); NM +  AI period (1970‒1989); AI +  ET period (1990‒2009); and finally, AI +  ET +  GS period (2010‒2021).

It is important to note that the SS and DS GIs decreased drastically from the beginning of the genomic era onwards, while the SD decreased gradually (Fig 2). This tendency changed again from 2017 onwards in both SS and DS paths, when the GI value increased again drastically (Fig 2).

### Pedigree Completeness and Genetic Diversity Evaluation

The pedigree completeness index (PCI) values of the reference Holstein-Friesian sire populations in different chronological periods are shown in Table 3. Overall, the PCI values increased over time. Comparison among the periods indicated that the PCI value between 1990 and 2009 (AI +  ET period) and between 2010‒2021 (AI +  ET +  GS period) were the highest, while the pedigree completeness between 1950 and 1969 (NM period) and between 1970 and 1989 (NM +  AI period) were the lowest (Table 3).

**Table 3:** Pedigree completeness-derived and inbreeding-derived parameters of the Ecuadorian Holstein-Friesian purebred males during the different chronological periods.

Chronological period	1950‒1969	1970‒1989	1990‒2009	2010‒2021
Number of animals in whole population	5,815	10,269	10,107	2,702
Number of animals in reference population	5,815	10,269	10,107	2,702
Number of inbred animals	204	5,578	9,315	2,622
Inbred animals (%)	3.51	54.32	92.16	97.04
Pedigre completeness índex (PCI; %)	32.88	55.44	78.71	92.97
Maximum generations traced	18	23	27	32
Mean equivalent generations	1.69	3.07	4.99	7.85
Complete generations	0.82	1.33	2.11	3.31
% known ancestors in:				
1^st^ generation	85.97	91.15	97.08	98.41
2^nd^ generation	45.51	74.34	91.93	97.66
3^th^ generation	19.39	53.63	82.02	94.71
4^th^ generation	9.05	35.62	68.72	90.18
Mean F (%)	0.19	0.82	2.49	4.41
ΔF (%)	0.08	0.22	0.47	0.55
Maximum coefficient of inbreeding (%)	31.25	37.62	38.41	30.62
Highly inbred animals ≥ 10% (%)	0.66	0.01	0.57	1.32
Average co-ancestry coefficient (C, %)	0.01	0.03	0.08	0.10
Average relatedness coefficient (ΔR, %)	0.11	0.61	1.68	2.19
Genetic Conservation index (GCI)	1.80	1.46	1.41	1.42
Ne (via individual increase in inbreeding)	163.08	186.29	102.93	89.14

Inbred animals: the animals with F >  0; Mean F: average inbreeding coefficient of all animals in reference population. ∆ F: the increase in inbreeding of all animals in reference population; Ne: effective population size.

**Fig 3:**
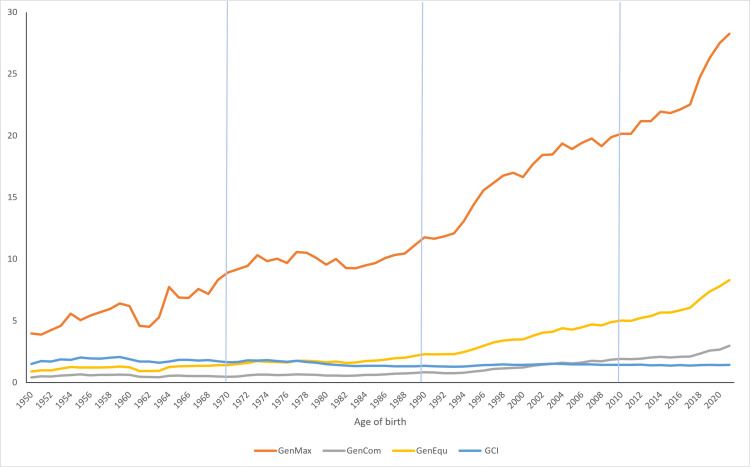
Genetic conservation-derived parameters in the Ecuadorian Holstein-Friesian purebred sires in the different chronological periods. Historical in-depth study of the genetic conservation indices of the Ecuadorian Holstein-Friesian purebred population. GenMax: maximum number of generations; GenCom: complete number of generations; GenEqu: equivalent number of generations; and finally, GCI: Genetic Conservation Index.

In addition, the maximum generations traced (GenMax) and mean equivalent generations (GenEqu) of the two last chronological periods were also higher compared to the other two periods (Fig 3). On the other hand, the analysis of known ancestors in different generations showed that in the first generation >  90% of the ancestors was known, except for the NM period ( ~ 85%). However, having into account just the fourth generation, only the third (AI +  ET) and the fourth (AI +  ET +  GS) period was ≥  70%, being the second (NM +  AI) period fallen below 50% and the PCI value less than 60%. Finally, the third (AI +  ET) and the fourth (AI +  ET +  GS) period exceeded PCI values of ~  80%. In summary, the pedigree completeness during the third (AI +  ET) and the fourth (AI +  ET +  GS) period was superior to those of the other periods. The requirements for pedigree integrity after the second (NM +  AI) period were also significantly higher than those before the same period.

### Inbreeding-derived parameters assessment

**Fig 4:**
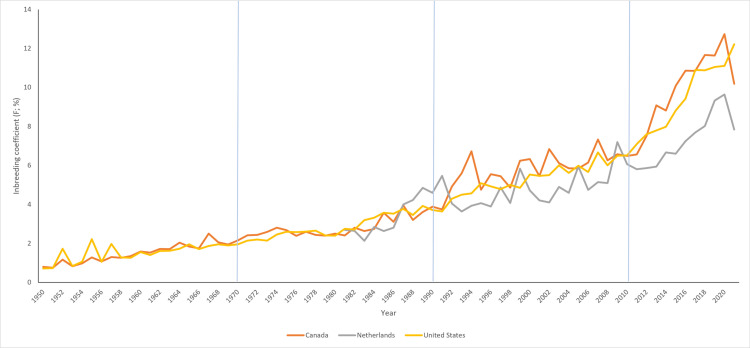
Inbreeding evaluation of the Ecuadorian Holstein-Friesian purebred males based on the most influential countries affecting genetic diversity from 2010 to 2021 (artificial insemination+  embryo transfer +  genomic selection period). Blue: United States; Brown: Canada; and Grey: Netherlands.

The inbreeding-derived and effective population size-derived parameters for each chronological period are shown in Table 3. The results showed that the proportion of inbred individuals of all periods has increased over time. Between 2010 and 2021 (AI +  ET +  GS period) the proportion of inbred individuals were significantly higher compared to the other three chronological periods (Table 3). However, although the proportion of inbreeding in the analyzed periods was high, the value of the mean inbreeding coefficient (Mean F) was quite moderate, ranging from 0.19% between 1950 and 1969 to 4.41% between 2010 and 2021. It is worthy to note that the reduced number of paternal lineages affected the GD of the Ecuadorian Holstein-Friesian cattle (Fig 4). In fact, the most utilized sires belonged to just three countries (USA, Canada, and Netherlands) which had a huge influence on the inbreeding values during the last decade (AI +  ET +  GS period).

**Fig 5:**
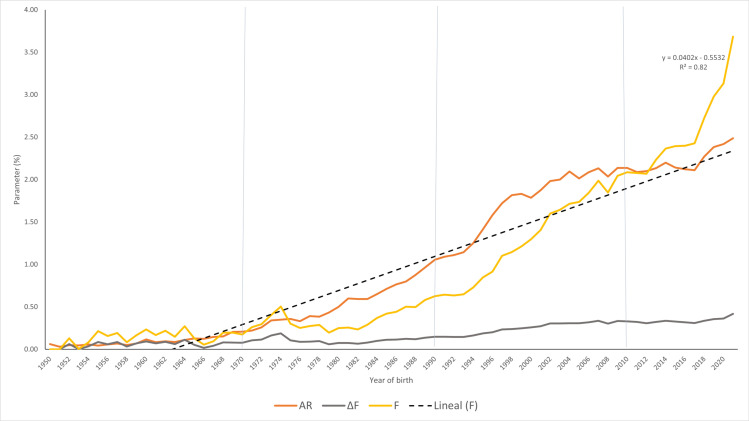
Inbreeding-derived parameters of the Ecuadorian Holstein-Friesian purebred sires during different chronological periods. Brown: Average relatedness coefficient (ΔR, %); Grey: Average individual increase in inbreeding (ΔF, %); Yellow: Inbreeding coefficient (F, %); Dot Line: Lineal inbreeding coefficient (F, %).

From the NM period till the occurrence of the genomic era, the average increase of inbreeding (∆F) showed an increasing trend from 0.08% to 0.55% (seven-fold increase) (Table 3; Fig 5). Similarly, the average relatedness coefficient (ΔR) increased thorough the chronological periods from 0.11% to 2.19%. It should be noted that the NM period was similar regarding ∆ F and ΔR parameters, remaining almost the same. However, both ∆ F and ΔR showed a dramatic increase from 1990 onwards. On the opposite, the tendency the related to the effective population size (Ne) showed a marked decrease (Table 3). Among the chronological periods studied, the AI +  ET +  GS period showed the lowest Ne, while the 1950‒1969 period (NM) and the 1970‒1989 period (NM +  AI) showed the highest Ne value.

### Gene origin probability

A summary of all parameters studied for the gene origin probability-derived analysis in different chronological periods are represented in Table 4. Among the studied periods, the highest number of founders (f) were detected between 1970 and 1989 (NM +  AI period) (9,131) and between 2010 and 2021 (AI +  ET +  GS period) (9,127); however, between 1950 and 1969 (NM period) and between 1990 and 2009 (AI +  ET period) a lower number of founders were observed being the NM period the lowest (4,865). Similarly, the same trend also was observed for the effective number of founders (fe) and the equivalent number of founders’ genomes (fge). The highest values of fe and fge were reached between 1970 and 1989 (NM +  AI period) (1,055) and between 1950 and 1969 (NM period) (860), respectively, and the lowest effective number of founders and founder genome equivalents were found between 1990 and 2009 (AI + ET period) 2010 and 2021 (AI +  ET +  GS period). However, it is worthy to note that the projection of future results may vary from these values because they belonged to an 11-year period. Overall, after the occurrence of the genomic era (2010-2021), the f, fe, and fge of the Holstein-Friesian breed sires decreased. In comparing different chronological periods, the fe/f ratio showed a similar trend between 1990 and 2009 (AI +  ET period) and between 2010 and 2021 (AI +  ET +  GS period); however, this ratio was higher between 1950 and 1969 (NM period) and between 1970 and 1989 (AI + ET period). Overall, the fge/fe ratio decreased over time (Table 4).

**Table 4:** Parameters derived from the gene origin probability and genetic diversity in Ecuadorian Holstein-Friesian purebred sires in the different chronological periods.

Chronological period	1950-1969	1970-1989	1990-2009	2010-2021
Number of ancestors	3,988	7,303	7,604	6,919
Total number of founders, f	4,865	9,131	6,523	9,127
Effective number of founders, fe	860	1,055	347	392
Founder genome equivalent, fge	214	61	28	18
fe/f ratio	0.18	0.12	0.05	0.04
fge/fe ratio	0.25	0.06	0.08	0.05
Number of ancestors to explain:				
25% of gene pool	35	9	4	3
50% of gene pool	267	75	31	15
75% of gene pool	1,239	903	655	138
100% of gene pool	3,988	7,303	7,604	6,919
GD	0.997	0.992	0.982	0.973
1-GD (GD loss)	0.002	0.008	0.018	0.027
DG^*^	0.9994	0.9995	0.9986	0.9987
Proportion of unequal contributions of the founders in GD loss (%)	24.89	5.76	8.08	4.70
Proportion of random genetic drift in GD loss (%)	75.11	94.24	91.92	95.30

GD: Genetic diversity. The probability of gene origin given by the effective number of founders; DG * : Genetic diversity in the reference population considered to compute the genetic diversity loss due to the unequal contribution of founders; (fe), total number of founders (f), and founder genome equivalents (fge).

Finally, the highest fge/fe ratio value occurred in the NM period (0.25) and the lowest value in the AI +  ET +  GS period (0.05) and the NM +  AI period (0.06). Overall, the fge/fe ratio decreased over time (Table 4).

**Fig 6:**
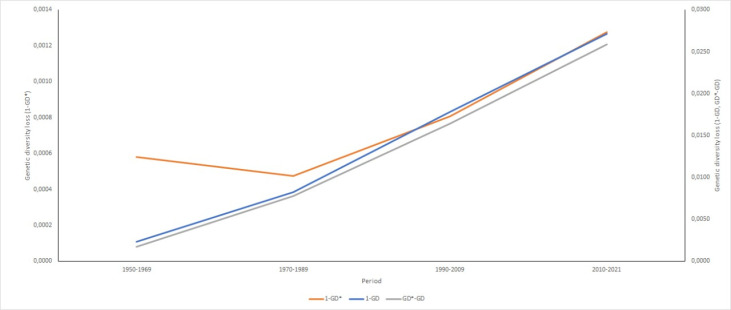
Genetic diversity (GD) loss of the Ecuadorian Holstein-Friesian purebred sires during different chronological periods. Left axis: GD loss due to unequal founder contributions (1 – GD*); Right axis: GD loss due to bottlenecks and genetic drift (1 – GD) and GD loss due to genetic drift only (GD * – GD).

The genetic diversity evolution for each chronological period is shown in detail in Fig 6.

The main cause of GD loss in Ecuadorian Holstein-Friesian sires was shown to be genetic drift accumulated over non-founder generations, which is mainly caused by the small Ne, irrespective of the chronological period studied. Moreover, unequal contribution of founders and bottlenecks jointly contributed relatively equal to the total loss during the NM +  AI period, AI +  ET period and AI +  ET +  GS period.

## Discussion

The present study reported for the first time the effects of paternal lineages on the genetic diversity as well as the inbreeding parameters and the effective size of the current population of Ecuadorian Holstein-Friesian sires. The aim of the present research was to generate base information to establish conservation strategies and reduce the effects of the GD loss in Ecuadorian Holstein-Friesian cattle populations. Overall, the number of paternal lineages was studied every 20 year from 1950 to 2021. The number of total males born increased from the 1950s to the 1990s; however, an inflection point was observed from the 1990s to the present, showing a drastic diminution in the males born. The number of modern era genomic-selected lineages that can be traced in the CDCB, among others, declined drastically over time maybe due to each generation was a subset of the parent generation. The number of generations traced during the later decades showed a better perception of this generation evolution in Ecuador. This was carried out comparing the number of paternal lineages over two generations for each chronological period taking into account the sires with adequate pedigree available. The obtained results suggested that paternal lineages were lost more rapidly as the adoption of AI together with the ET technologies became widespread. All three lineages trace their paternal ancestry to sires from which semen was imported from United States, Canada, and Netherlands. The three dominant modern ET era together with genomic lineages account for >  90% of all Ecuadorian sires born in the 1990s, 2000s, and 2010s. Despite being responsible for almost all current paternal lineages descendants, the three paternal lineages were relatively unrelated to the female Holstein-Friesian population [9,10]. This indicates that, although the three paternal lineages were responsible for almost all male lineages, other sires from the modern ET and genomic era contributed with new genetic merit through female lineages. The PTA values for fat and protein yields were higher for the parental lineages during the 2010s compared to the paternal lineages during the 1990s and 2000s [8,27,29,32]. Therefore, some of the older lineages could be lost in the following years. However, the presence of the modern lineages demonstrates the high influence of embryo transfer technologies and genomic selection in enhancing GD based on specific sires [17,18]. Of those sires born in United States in the 1990s, whose were submitted to an official evaluation, the paternal lineage was difficult to trace further. This suggests that there is limited GD based on paternal lineages, but there is evidence of new lineages during ET technologies and genomic selection era based on different maternal lineages.

From 1950 onward, the number of born males increased because Ecuador began to import semen mainly from USA, Canada, Netherlands and New Zealand for AI purposes [16]. However, from 1990 onward the development of new ARTs such as ET and AI using sex-sorted semen, and genomic selection led to decrease the number of sires born, together with the decrease of the number of sires imported, and then, the population became closed [16,30]. Thus, in the present study the official pedigree records were analyzed and several parameters such as F, Ne, fe, and fge were obtained to assess the population GD during four chronological periods with the aim of understanding the impact of these periods on the GD. The accuracy of indicators such as F, Ne, and GD depends on the pedigree quality of the population [47]. The present research showed that the PCI value from 1970 onward reached more than 55%, and the percentage of known ancestors in the fourth generation from 1990 onward was higher than ~ 70% indicating a reasonable estimation of other pedigree indicators. In addition, the correlated variables of pedigree integrity of each population before and after 1990 (arrival of new ARTs) was very similar enhancing the subsequent variable comparisons. Although the pedigree quality results of the Ecuadorian Holstein-Friesian population is moderate compared to some recent pedigree analyses of Holstein-Friesian sires in other countries [48], the pedigree completeness in the present study was similar to those reported before in American and Canadian sires [49,50] and worse than other previous studies [51]. These differences in the pedigree quality indicators between Holstein-Friesian sires found in the present research might be caused by the dynamics and intensity of individual sires used in commercial breeding programs in different countries [52]. Moreover, other differences could be attributed to the impact of specific imports or the depth of pedigree knowledge of imported sires [53]. Thus, these indicators would affect the pedigree quality, and therefore, the estimated inbreeding and GD [21,54]. Overall, the GIs observed in sires in the present research were slightly shorter than those reported on dams [55]. Similar results were found in previous studies [2,3,6]. Regarding GI, the periods before 1990s showed an increasing trend in the present research. However, after 1990s the trend changed drastically showing a decreasing GI pattern. Overall, the GI of all gametic pathways decreased over time. This fact may be because after the inclusion of sex-sorted semen and ET technology and that the Ecuadorian breeding cattle industry increased the semen and embryo importation [12]. As a consequence, the demand for breeding stock increased in favor of the purebred population which was focused in certain elite sires [56]. This could be explained by the younger breeding age of sires and dams in the herds from 1990s onwards due to the breeders increased the confidence in using genetic material from new breeding sires rather than keeping the current breeding stocks allowing new generation to join, especially in the sire-son path of each chronological period from 1990s onwards. This tendency was improved through on-farm dairy production testing, which has gradually improved in recent years in Ecuador [2]. By performing production, reproduction and genetic performance testing assays it was possible establishing different breeding selection indices of Holstein-Friesian purebred progeny identifying the most appropriate sires for mating them at younger age shortening the GI and reducing the breeding costs [57,58]. The obtained ∆ F values over the periods evaluated in the present study were similar to those of previous studies [49] indicating a ∆ F value for the American Holstein-Friesian cattle of approximately 0.17-0.22%, while for the Canadian Holstein-Friesian was around 0.08-0.29% [21]. With regard to other Canadian dairy cattle breeds the ∆ F values per decade were around 0.44-1.52% [50] Moreover, the inbreeding degree of the Ecuadorian Holstein-Friesian cattle was lower than the Brazilian or Mexican [59,60,61]. The increase in inbreeding of all animals in reference population (∆F) cold indirectly be an efficient indicator for measuring the Holstein-Friesian population health, being a value of ∆ F <  1% considered as a reasonable rate according to the Food and Agriculture Organization of the United Nations, [62,63]. In the present study, all ∆ F rates obtained during the different periods were below the purposed threshold. Regarding the effective population size (Ne) it should be maintained > 50 in order to withstand the effects of F value according to the FAO [62,63]. In the present research, the Ne observed during the analyzed periods was above this value; however, a size of Ne ≥ 500 is crucial to guarantee the genetic variability and diversity of the population for several generations [3,64]. In our study, the current and past Ne values in Ecuadorian Holstein-Friesian were well below this threshold. Therefore, there is a pressing need to reestablish the Ne values in order to construct a more varied Holstein-Friesian breed population [23]. Although several authors reported similar Ne values than in the present research it just shows a general problem based on the lack of genetic variability in Holstein-Friesian cattle in different countries [65,66]. During the last years, the use of the genome information from 2010 onwards in Ecuador helped for the improving the selection schemes based on the GD evaluation [67]. The genomic-derived information together with the inbreeding coefficients could be successfully used as an allelic diversity predictor which could be applied to the breeding selection schemes [68]. The results obtained from the genomic analyses may be used as complementary information to the pedigree datasets making them more comprehensive, complete, and accurate to discover the reason of GD loss observed in the present study and to find solutions to resolve it. The Ne value increased from the 1950s to the 1990s, but decreased with time from the 1990s to the 2020s, being reduced to a critical Ne value <  90, closer and closer to the limit value (Ne = 50). Moreover, the last period (2010 to 2021) showed the greatest inbreeding coefficient. According to the observed results, the dairy industry needs to develop a new strategy for improving the existing population by import new genetic resources before multiplying the existing herd with the aim of increasing the ∆ F rate which was very limited in the Ecuadorian Holstein-Friesian breed. An important issue to be considered is that ARTs, including AI and ET can quickly reduce the GD, and therefore, the application of ARTs to extremely specialized dairy cattle such as Holstein-Friesian breed requires a rigorous control avoiding the GD loss in a given population [24]. In the present study an apparent steady trend of GD was observed over the periods. The gene origin during the studied periods required a quite low number of ancestors to be explained; however, in the present study the number of ancestors to explain the gene pool is much lower than those presented in other countries based on Holstein-Friesian populations [69,70]. An important factor that should be taken into account to explain the reasons of GD loss is related to the fe/f ratio that was able to explain the GD loss based on the founders who showed an unequal contribution. Moreover, the influence of the genetic drift on GD can be explained, at least in part, by the fge/fe ratio. On the other hand, if the values of the effective number of founders (fe) and the total number of founders (f) were similar would mean that all founders would contribute similarly. In the present study, the value of fe and f was unequal indicating that there was a genetic selection factor which was definite and very influential on the founder contributions [71]. This fact is a consequence of the breeding selection schemes carried out by the breeders whose have been choosing the same sires during decades unbalancing the founder’s ratio in the Holstein-Friesian cattle, as showed in the present study. Traditionally the selection intensity in the Holstein-Friesian cattle was based on the average daily milk production records avoiding reproductive and longevity-derived traits [72]. The great fge/fe ratio observed in the different chronological periods indicates the notable effect of genetic selection over time, especially in the two last periods studied. Previous studies on Holstein-Friesian populations of USA and Canada showed the consequences of the genetic selection [68]. The genetic drift was also identified as the main cause of GD loss in different dairy cattle breeds in Canada [50] and Japanese black cattle [46]. In this case the GD loss it was related random genetic drift over the non-founder generations. In the present study, the lack of genetic drift was evident over time affecting directly the GD which indicates the dramatic decline of the Holstein-Friesian population derived from the arrival of new ARTs from 1990s onwards together with the genomic selection during the last period increasing the homozygosity and fixation of alleles in Holstein-Friesian cattle [32]. Therefore, the introduction of new genetic resources and the application of controlled breeding schemes for the Ecuadorian Holstein-Friesian cattle are advised, as happens with other cattle breeds raised in Ecuador [73], with the aim of reducing the inbreeding parameters and increasing the genetic variability and GD of the herds [ 74,75].

## Conclusions

In summary, based on the information derived from the progeny tests the Ecuadorian Holstein-Friesian cattle should be managed to mate the new generation sires at a younger age with the aim of reducing the GI. The GD-derived parameters showed that after the 1990s with the arrival of new ARTs and genomic selection schemes triggered the GD loss in the Ecuadorian Holstein-Friesian cattle. Currently it should be a priority in Ecuador to reduce the inbreeding rates introducing unrelated genetic material and re-formulate new breeding selection and mating schemes increasing the Ne of Holstein-Friesian sires. Moreover, the ∆ F rate has been increased drastically over time. Finally, the breeding schemes should be performed taking into account the Holstein-Friesian cattle able to be adapted to the Ecuadorian environmental conditions using alternately the foreign genetic resources and the national breeding stock with the aim of reducing F values and increasing the GD in the current Ecuadorian Holstein-Friesian population.

## Abbreviations

GD: Genetic diversity
Ne: Effective population size
NM: Natural mating
AI: artificial insemination
ET: Embryo transfer
GS: genomic selection
EHFA: Ecuadorian Holstein-Friesian Association
CDCB: Council Dairy Cattle Breeding
CNDE: Canadian Network for Dairy Excellence
BLUP: BEST linear unbiased prediction
MACE: Multiple Across Country Evaluation
ARTs: Assisted reproductive technologies
MOET: Multiovulation and embryo transfer
SS: Sire-son
SD: Sire-daughter
DS: Dam-son
DD: Dam-daughter
ΔR: Genomic relatedness
F: Inbreeding coefficient
C: Co-ancestry coefficient
AR: Average relatedness coefficient
∆ F: Individual inbreeding rate
∆ C: Individual co-ancestry rate
GCI: genetic conservation index
GenMax: Maximum number of generations
GenCom: Complete number of generations
GenEqu: Equivalent number of generations
GI: Generation interval
f: total number of founders
fe: Effective number of founders
fge: founder genome equivalents
Fped: Pedigree-based inbreeding
Fgen: Genomic-based inbreeding
TPI: Total performance index
NM$: Net merit
MLK_ PTA: Predicted transmitting abilities of milk
FAT_PTA: Predicted transmitting abilities of fat
PRO_PTA: Predicted transmitting abilities of protein
PL_PTA: Predicted transmitting abilities of productive life
DPR_PTA: Predicted transmitting abilities of daughter pregnancy rate
